# Choledochal Cyst Mimicking Gallbladder with Stones in a Six-Year-Old with Right-sided Abdominal Pain

**DOI:** 10.5811/westjem.2015.4.25407

**Published:** 2015-06-22

**Authors:** Rachna Subramony, Nat Kittisarapong, Isabel Barata, Matthew Nelson

**Affiliations:** * University of Massachusetts, Department of Emergency Medicine, Boston, Massachusetts; † Northshore University Hospital, Department of Emergency Medicine, Manhasset, New York

## Abstract

Choledochal cysts are rare but serious bile duct abnormalities are found in young children, usually during the first year of life.^1^ They require urgent surgical intervention due to the risk of developing cholangiocarcinoma.^2^ Clinicians should consider this diagnosis and perform a point-of-care ultrasound (POCUS) when a child presents to the emergency department (ED) with findings of jaundice, abdominal pain, and the presence of an abdominal mass. We present the case of a six-year-old child presenting only with abdominal pain upon arrival to our ED and was ultimately diagnosed by POCUS to have a choledochal cyst.

## INTRODUCTION

Choledochal cysts are rare congenital abnormalities usually found in infants and children. They require urgent surgical intervention due to a 10% risk of cholangiocarcinoma.^2^ These cysts are developmental anomalies in which there is a disproportionate dilatation of the biliary ductal system.^3^ The incidence of choledochal cysts averages one in two million births with a three times greater predominance in females. Over 60% are diagnosed during the first year of life.^1^ The classic triad includes pain, jaundice, and the presence of an abdominal mass; however less than 20% of patients will have all three findings. In addition, there are no specific laboratory tests to help identify these cysts, making it even more of a challenging emergency department (ED) diagnosis.^4^ We present a case of a six-year-old patient with a choledochal cyst, identified on ultrasound in the ED.

## CASE REPORT

A six-year-old female of Asian descent born full term by spontaneous vaginal delivery with no past medical or surgical history presented to the ED with right-sided abdominal pain that started five days prior to presentation. The patient described the pain as sharp, intermittent, and located in the right upper quadrant and epigastric region of the abdomen without any aggravating or alleviating factors. She had a decreased appetite, constipation, and fatigue. However, she and her family denied nausea, vomiting, changes in stool or urine color, fevers, or weight loss. The patient was seen by her pediatrician earlier that day and had been sent to the ED for evaluation of possible appendicitis.

Her physical examination revealed the following vital signs: pulse rate of 138 beats per minute; respiration rate of 20 breaths per minute; temperature of 35.9 degrees Celsius orally; oxygen saturation of 98% on room air; and weight of 22.6kg. The patient was alert, well appearing, and in no apparent distress. The head, eyes, ears, nose, and throat exam was unremarkable. The heart sounds were normal and the lungs were clear. The abdomen was soft, with normal bowel sounds, and mild tenderness in the right upper quadrant with no rebound, guarding, or radiation. No masses or hernias were appreciated. The spleen and liver were normal in size. No lymphadenopathy was appreciated. Gait was normal with mild abdominal discomfort appreciated when the child was asked to jump. Skin was normal with no rashes or jaundice. The neurologic exam was within normal limits.

**Figure 1:**
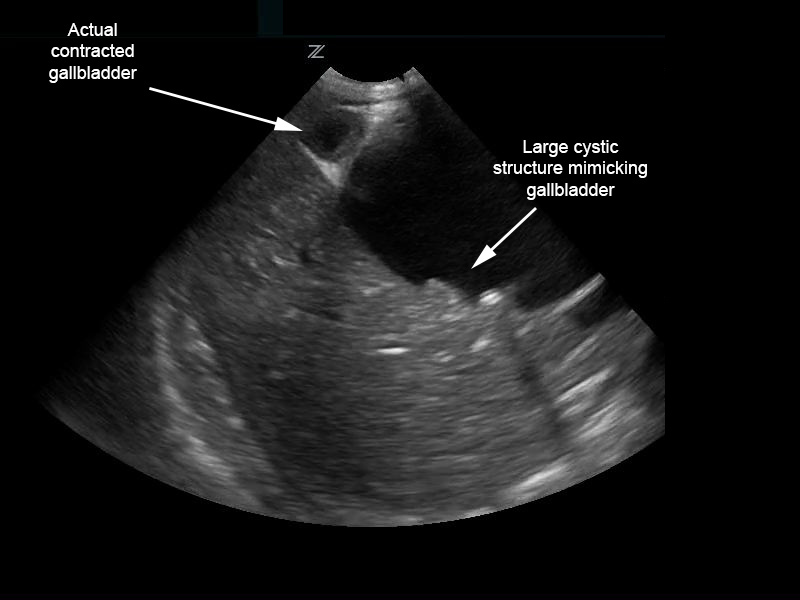
Biliary choledochal cyst mimicking distended gallbladder with multiple stones; however, contracted gallbladder is actually adjacent.

**Figure 2:**
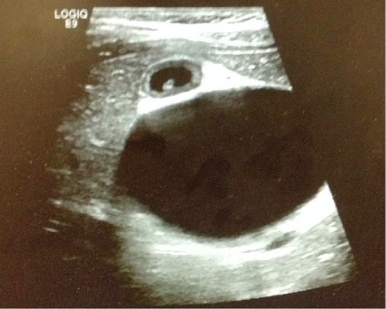
Follow-up radiologic ultrasound showing presence of mild intrahepatic biliary ductal dilatation as well as a large cystic mass with internal echoes and debris confirmed as a choledochal cyst. The gallbladder with multiple gallbladder stones or polyps is also pictured above.

The patient’s urinalysis was normal, and an ultrasound was performed at the bedside using a low frequency (2–6 MHz), curvilinear probe oriented in a transverse then sagittal plane fanning superiorly then inferiorly, medially then laterally along the child’s right upper quadrant along her subcostal margin. The point-of-care ultrasound (POCUS) demonstrated what initially appeared to be a distended gallbladder with shadowing stones (Figure 1). However, with continued scanning, a small contracted gallbladder with stones versus polyps was identified adjacent to the originally visualized larger cystic structure. A complete abdominal ultrasound performed by the radiology department showed normal echogenicity and echotexture of the liver with no masses and a small amount of perihepatic free fluid. There was mild intrahepatic biliary ductal dilatation due to a large cystic mass with internal echoes and debris measuring 6.4×5.1×5.3cm and originating from the common bile duct (CBD). The gallbladder was visualized with multiple gallbladder stones or polyps, the largest measuring 4mm (Figure 2). The pancreas, spleen, and kidneys appeared unremarkable and the appendix was not visualized; however, a small amount of free fluid was seen in the right lower quadrant. Based on this sonographic appearance, the diagnosis of choledochal cyst was made.

After the POCUS was performed the patient was transferred to the children’s hospital for surgical management and an magnetic resonance imaging of the abdomen that showed findings similar to the ultrasound. The CBD cyst measured 6.7×4.8×8.0cm and extended from the porta hepatis to the pancreatic head. There were multiple small stones in the cyst but no solid components. The extrahepatic portal vein was mildly deviated to the left due to a mass effect. There was mild gallbladder dilatation with stones. The remainder of the study was negative. These results were consistent with a Todani type Ic bile duct cyst. The patient underwent open resection of the choledochal cyst, cholecystectomy, and a hepaticoduodenostomy biliary bypass. The patient tolerated the procedure well, was extubated shortly after surgery, and was discharged home six days after surgery.

Final pathology report showed that the cystic tissue had rough fibrous connective tissue and friable mucosa consistent with reactive changes. The gallbladder tissue was smooth with a congested dark red serosal surface consistent with chronic cholecystitis. At the two-month follow up, the patient had regained her appetite, was doing very well, and was pain free.

## DISCUSSION

While choledochal cysts are most commonly found in children under the age of 10, up to 20% are diagnosed at adulthood.^1^ Some cysts have been detected before birth by prenatal ultrasounds as early as 15 weeks. While some believe these cysts are congenital, there have also been reports of new cysts diagnosed in patients older than 80. Overall these cysts are believed to be as rare as one in two million births with a 4:1 predominance in females.^5^ The incidence is higher in the Asian population of which about two-thirds of cases are reported from Japan.^6^

Choledochal cysts are generally classified into five subtypes by the Todani classification developed in 1977. Since then, several other classification systems have been developed, but Todani’s is still widely accepted. Type 1 cysts are extrahepatic cysts in the CBD. These are further subdivided into types a-c. A type 1a cyst consists of diffuse CBD dilatation, a type 1b cyst has focal segmental dilatation, and a type 1c has fusiform dilatation of the CBD as in our patient. Type 2 cysts are extrahepatic duct diverticula. Type 3 cysts are intraduodenal or intrapancreatic ductal dilatations. Type 4 cysts are multiple bile duct cysts and type 5 cysts are intrahepatic cysts also referred to as Caroli disease.^3^

There is no definite explanation for the pathogenesis of these cysts; however, multiple studies suggest they may be formed due to prolonged reflux of pancreatic enzymes and bile stasis leading to chronic inflammation.^7^–^8^ This chronic inflammation may be one factor responsible for the development of cholangiocarcinoma in patients with bile duct cysts. Other factors shown to cause malignant transformation of these cysts include cell regeneration and DNA dysplasia.^2^ Cystic fluid after removal has also been found to have an elevated amylase and lipase in up to 65% of cases^8^ and pancreatic reflux can cause K-ras mutations and cellular atypia leading to carcinogenesis. The incidence of cholangiocarcinoma in patients with choledochal cysts ranges from 2.5% to 26% with an increasing risk with age.^2^

Malignant changes in the epithelium of the bile duct can occur even after cyst excision, sometimes years after cyst removal. These patients therefore need to be followed for recurrent symptoms. Some of these malignant transformations have been seen in areas distant from the CBD cyst.^9^ Common locations for developing malignancy are the bile duct in 50% of patients, the gallbladder in 43%, and the periampullary region in 2.5%.^10^

Classic findings include jaundice, pain, and an abdominal mass; however, less than 20% of patients will have all three findings. Classic findings are more common at a younger age with 85% of children having at least two of the three findings and only 25% of adults having two out of three findings. Laboratory results including liver enzymes are generally normal.^4^

Because of the lack of radiation, abdominal ultrasound should be the primary imaging modality used in young children with right upper quadrant pain presenting to the ED. Ultrasound has been shown to be superior to computed tomography (CT) in identifying gallbladder pathology and avoids exposing the patient to unnecessary radiation. Emergency bedside ultrasound by trained physicians has both a sensitivity and specificity of greater than 88% for detecting gallbladder pathology.^11^ It has a sensitivity of 71–97% specifically for choledochal cyst detection.^12^ Other modalities that have been shown to be highly sensitive in the diagnosis of biliary cysts include hepatobiliary scintigraphy using technitium-99 hepatobiliary iminodiacetic acid (HIDA scan), which has a sensitivity of 100% for type I bile duct cysts, and magnetic resonance cholangiopancreatography, which is the most sensitive diagnostic modality.^12^ CT and magnetic resonance imaging are helpful after diagnosis to help create a management and treatment plan. Due to the high rate of malignancy all biliary duct cysts require surgical removal.

Historically, cystenterostomy was considered the best surgical treatment for patients with choledochal cysts. However, this approach was found to be associated with a high risk of malignancy in the remaining cyst wall with up to 30% of adults developing malignancy after cyst removal.^13^ Therefore, biliary diversion after excision is now the recommended surgical treatment and can be done by hepaticoduodenostomy as in our patient, or via hepaticoappendicoduodenostomy, or hepaticojejunostomy.

Because a choledochal cyst is a serious condition in which a delay in diagnosis can lead to a worse clinical outcome, young patients suspected of having this condition should undergo an abdominal ultrasound and if a choledochal cyst is found, the patient should have early surgical removal in order to avoid complications and prevent malignancy. Thus, emergency point-of-care ultrasound as a first-line diagnostic tool may prove to be a life-saving measure in detecting rare but potentially fatal pediatric abnormalities such as a choledochal cyst.

## Figures and Tables

**Video:** Point of care ultrasound for Figure 1.
